# Wells Syndrome: A Rare Skin Manifestation of Non-Hodgkin's Lymphoma

**DOI:** 10.7759/cureus.71150

**Published:** 2024-10-09

**Authors:** Akarsh Jose, Mithun Raj, Manoj P Jose, Jayasree MG

**Affiliations:** 1 Internal Medicine, Little Flower Hospital and Research Centre, Angamaly, IND; 2 Gastroenterology, Little Flower Hospital and Research Centre, Angamaly, IND; 3 Pathology, Lakeshore Hospital and Research Centre, Ernakulam, IND

**Keywords:** lymph node, new-onset jaundice, non-hodgkin’s lymphomas, skin lesions, wells syndrome

## Abstract

Eosinophilic cellulitis, also known as Wells syndrome, presents a wide range of morphological spectrum, from pruritic erythematous papules, nodules, and pustules to urticarial and bullous lesions. This is a rare dermatological condition and is known to develop after treatment of hematological malignancy. Here, we report a case of Wells syndrome that was the initial presentation of lymphoma, preceding all other symptoms by six months.

## Introduction

The syndrome was first described in 1971 by George Crichton Wells as recurrent granuloma­tous dermatitis with eosinophilia. The condition was renamed eosinophilic cellulitis and Wells syndrome in 1979 [1]. Wells syndrome is an infrequent dermatological condition whose morphological type ranges from typical cellulitis-like lesions to rare bullous vesicular lesions [2]. The exact etiology of Wells syndrome is still unknown, but there are a few conditions associated with it like infections, allergies, and myeloproliferative diseases [3]. This case report describes diffuse skin lesions all over the body as an early presentation of non-Hodgkin’s lymphoma.

## Case presentation

**Figure 1:**
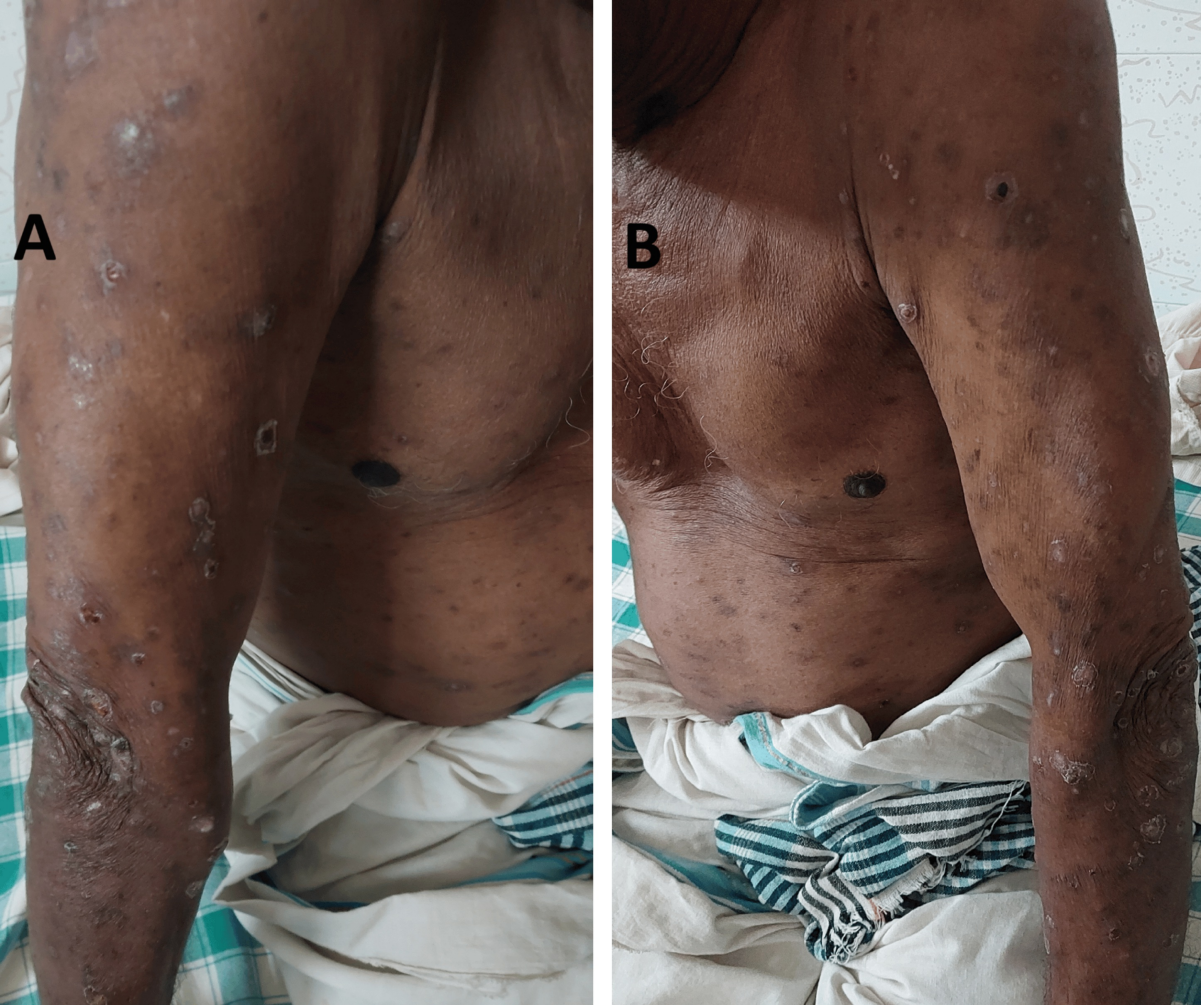
Skins lesions on presentation to hospital. (A, B) Papular and nodular lesions seen on chest, abdomen, and upper limbs

A 73-year-old male patient with no comorbidities presented with severe pruritic rashes all over the body for six months. The patient also reported a fever in the last two weeks and dark-colored urine on the day of presentation to the hospital. Pruritic rashes were initially seen over the chest and abdomen as reddish papules and nodules, spreading to his extremities and face (Figure 1). Scalp, palms, and soles were spared. Purulent discharge was present from the skin lesions.

On examination, the patient appeared icteric and had bilateral pitting pedal edema. Generalized lymphadenopathy with bilateral involvement of cervical, axillary, and inguinal lymph nodes along with right supraclavicular and right epi-trochlear node was present. Lymph nodes in all these areas were non-tender, firm, rubbery in consistency, and mobile. The patient was febrile with a temperature of 101^o^F, pulse rate of 76/minute, and blood pressure of 130/80 mmHg. Diffuse erythematous papulo-nodular skin lesions affecting the trunk, limbs, and face were present. Oral and genital mucosa, scalp, palms, and soles were unaffected. Purulent discharge and crusting were noted in some lesions. Examination of the abdomen revealed a non-tender, firm liver palpable 8 cm below the right costal margin in the mid-clavicular line with a rounded edge and liver span of 18 cm. A firm, non-tender spleen was palpated 3 cm below the left costal margin. 

Investigations showed normocytic anemia, with eosinophilia and elevated erythrocyte sedimentation rate (ESR). The liver function test was deranged with elevated bilirubin, transaminases, and alkaline phosphatase. Hyponatremia, hypoalbuminemia, and elevated prothrombin test time and international normalized ratio (INR) were observed. Elevated lactate dehydrogenase (LDH), serum ferritin, and C-reactive protein (CRP) were also noted (Table 1).

**Table 1:** Laboratory results

Tests	Patient Values	Normal range
Hemoglobin	9.2 g/dl	12.5 - 17 g/dl
White cell count	6000 cells/mm^3^	4000 - 11000 cells/mm^3^
Neutrophils	66%	50 - 70%
Lymphocytes	12%	25 - 40%
Monocytes	7%	2 - 8%
Eosinophils	15%	1 - 6%
Platelet count	274000 /microliter	150000 - 450000 /microliter
Erythrocyte sedimentation ratio (ESR)	105	0 - 45mm/hr
Total bilirubin	6.94 mg/dl	0.2 - 1.2 mg/dl
Direct bilirubin	4.13 mg/dl	0 - 0.3 mg/dl
Aspartate aminotransferase (AST)	292 U/L	5 - 50 U/L
Alanine aminotransferase (ALT)	170 U/L	5 - 50 U/L
Alkaline phosphatase	1402 U/L	30 - 120 U/L
Total protein	5.8 g/dl	6.4 - 8.3 g/dl
S. Albumin	3.0 g/dl	1.8 - 3.6 g/dl
Albumin: Globulin Ratio	1	1.1- 2.2
Serum Urea	28 mg/dl	10 - 43 mg/dl
Serum Creatinine	1.10 mg/dl	0.6 - 1.44 mg/dl
Serum Sodium	120 mEq/L	135 - 150 mEq/L
Serum Potassium	4.4 mEq/L	3.5 - 5.5 mEq/L
Serum Calcium	8.0mg/dl	8.6 - 0.6mg/dl
Prothrombin time	15.8 seconds	10 - 13 seconds
International normalized ration (INR)	1.47	0.8 - 1.2
C-reactive protein (CRP)	75.95 mg/L	0 - 6 mg/L
Lactate dehydrogenase (LDH)	581 U/L	5 - 247 U/L
Serum Ferritin	1611.0 ng/ml	25 - 380 ng/ml
Dengue IgM Antibodies	Negative	-
Leptospirosis IgM Antibodies	Negative	-
Brucella IgM Antibodies	Negative	-

Chest X-ray showed bilateral prominent hilum and para-tracheal shadows. Ultrasonography of the abdomen confirmed hepatomegaly, splenomegaly, and the presence of minimal ascites. Multiple lymph nodes of varying size with altered corticomedullary differentiation were also visible in ultrasonography. Contrast-enhanced CT of the abdomen showed multiple discrete homogenously enhancing lymph nodes in peri-portal, peri-pancreatic, pre-aortic, para-aortic, aortocaval, and retrocaval areas, and bilateral inguinal lymphadenopathy in addition to hepatomegaly and splenomegaly. Contrast-enhanced CT of the chest revealed multiple discrete homogenously enhancing lymph nodes in pre-tracheal, para-tracheal, pre-carinal, sub-carinal, para-oesophageal regions, and bilateral axillary lymphadenopathy.

**Figure 2:**
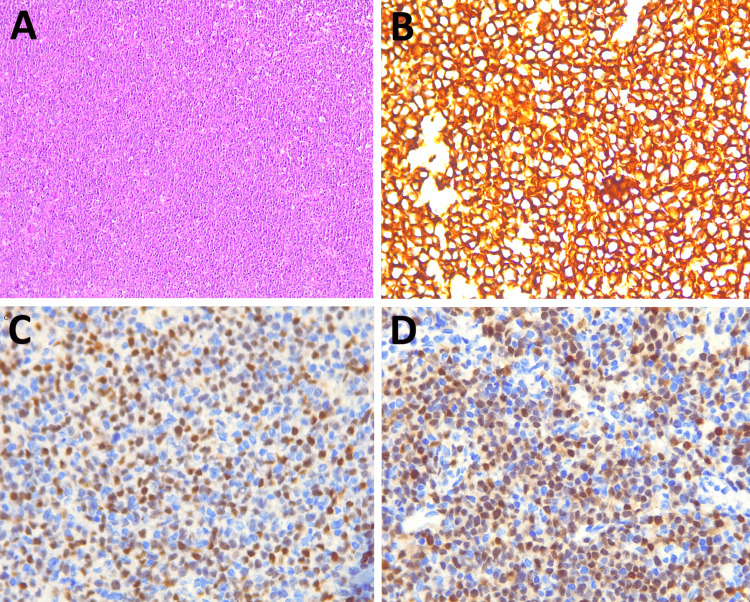
- Histology and immunohistochemical staining of lymph node (A) Lymph node architecture effaced by diffuse and nodular infiltrate of large cells; (B) Positive for CD 20; (C) Positive for c-Myc; (D) Positive for MUM1

**Figure 3:**
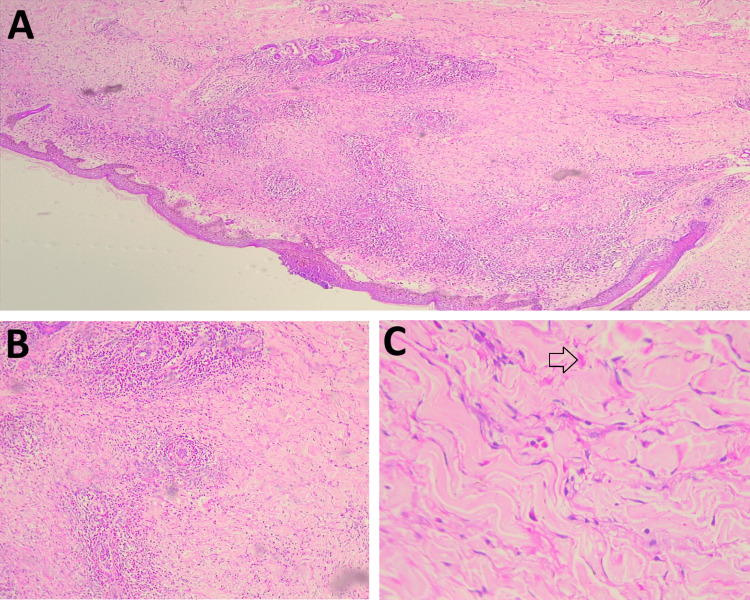
Histology of skin biopsy (A) Sub-epidermal separation and dermal eosinophilic rich reaction; (B) Perivascular eosinophilic-rich inflammation on medium power magnification; (C) The arrow marked shows flame figure on high power magnification

Skin and cervical lymph node biopsies were taken. GeneXpert (Cepheid, Sunnyvale, California, United States) for *Mycobacterium tuberculosis* and fungal culture were negative. Lymph node biopsy showed lymph node architecture effaced by diffuse and nodular infiltrate of large cells with high mitotic activity suggestive of diffuse large B cell lymphoma (Figure 2A). Immunohistochemistry on the cervical lymph node showed lymphomatous infiltrate positive for CD20, c-Myc (90%), and MUM 1(90%) with a Ki 67 index of 85-90% (Figures 2B, 2C, 2D). Skin biopsy showed sub-epidermal separation and marked dermal edema (Figure 3A). Eosinophilic infiltrates around vessels and flame figures suggest Wells syndrome (Figures 3B, 3C).

**Figure 4:**
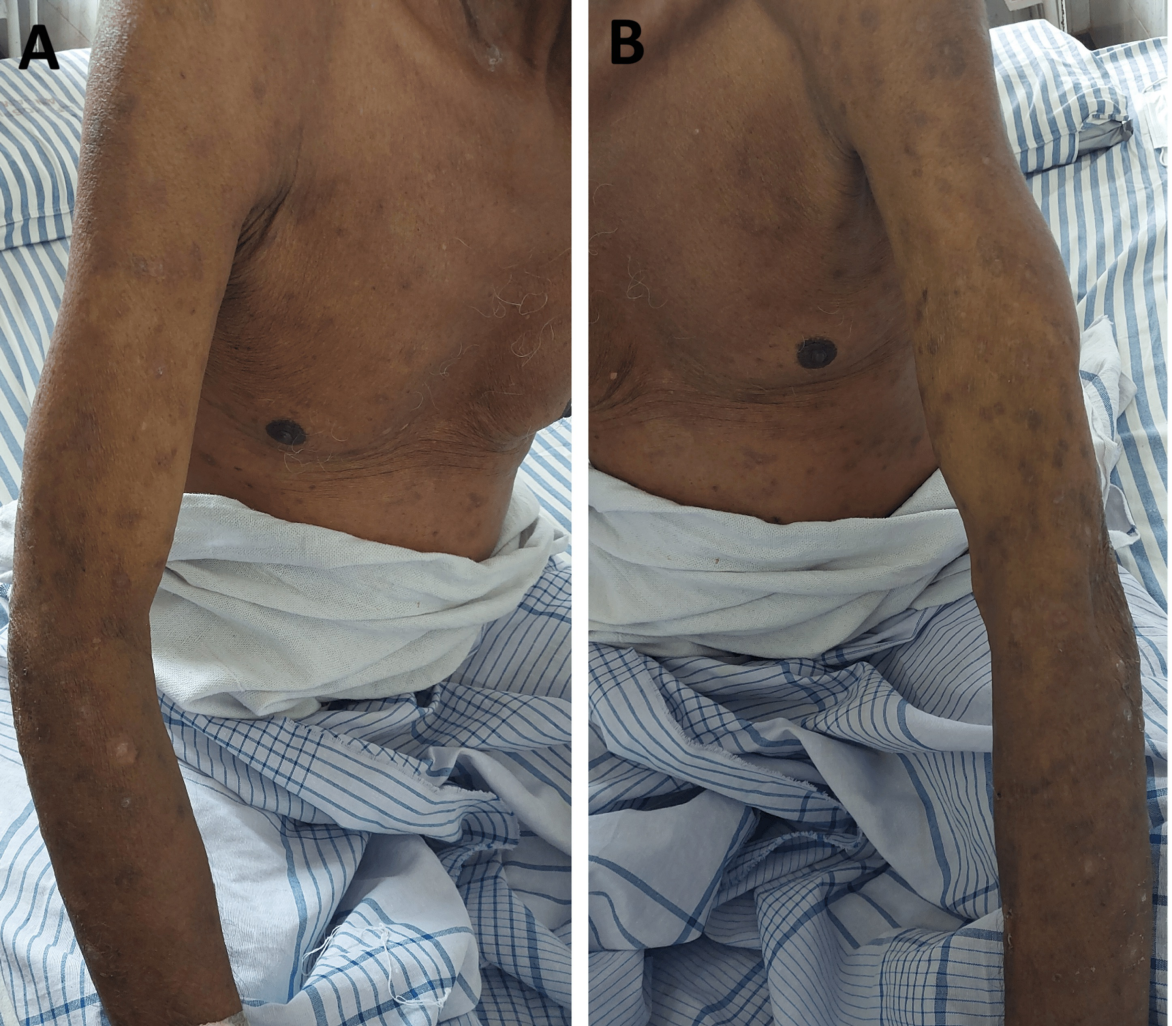
Resolution of skin lesions with treatment

A diagnosis of diffuse large B-cell lymphoma with Wells syndrome was made, and the patient was started on steroids (tab. prednisolone 40 mg per day) and antihistamines. Existing lesions resolved, and no new lesions appeared after commencing the treatment (Figure 4). The patient was referred to the oncology department for further treatment of non-Hodgkin’s lymphoma.

## Discussion

Wells syndrome is a rare chronic inflammatory dermatitis characterized by its clinical polymorphism. The etiology and pathogenesis of this condition are still under study. It presents typically as red-violet erythematous papules, nodules, plaques, or rarely bullous lesions with intense pruritus [2]. These skin manifestations are often associated with peripheral eosinophilia and usually return to normal levels in cases of clinical remission with treatment [2,4].

Histopathological features of Wells syndrome have been classified into the following stages: (i) an acute or eosinophilic cellulitis stage which shows edema and dense infiltration of eosinophils and histiocytes into the dermis; (ii) a subacute or granulomatous dermatitis stage in which eosinophils get adhere to collagen fibers associated with degranulation and thus forming the characteristic flame figures and (iii) a resolution or atrophy stage demonstrating few eosinophils, giant cells associated with foreign body, and histiocytes [5].

Numerous triggering or associated factors of Wells syndrome have been identified, which include infections (type 2 herpes simplex virus) [6], some parasites, insect bites or stings, lymphoproliferative diseases [7], hematological malignancies chronic myelogenous leukemia (CML) [8], chronic lymphocytic leukemia (CLL) [9], solid tumors [10], vasculitis like Churg-Strauss syndrome, and chronic inflammatory diseases (ulcerative colitis). There is literature on documented cases associated with using immunosuppressive drugs infliximab [11] and adalimumab [12]. Wells syndrome is hypothesized to be a hypersensitivity reaction to various exogenous and endogenous antigens. Histopathology showed eosinophilia and a high proportion of CD4+ cells by producing IL-5.

A study of a series of cases of idiopathic Wells syndrome found that oral steroids were the most effective treatment choice, with a success rate of about 92%. However, treating the underlying cause of Wells syndrome is also very important if any such cause is found, and alternate-day low-dose oral steroids are advised in recurrent cases [13].

In this case, the skin lesions developed months before other symptoms, and an evaluation of skin lesions helped us to reveal the underlying diagnosis of non-Hodgkin’s lymphoma. So, a clinical and laboratory assessment for evidence of any lymphoproliferative disorders should be considered a vital step in the diagnostic work-up of refractory skin lesions because there are reports of likely association between skin lesions and lymphoproliferative diseases [7]. Treatment with systemic corticosteroids and antihistamines helped resolve skin lesions in this patient.

## Conclusions

Although no underlying disease process can be found in most cases of Wells syndrome, it has occasionally been associated with hematological malignancies. Still, it is typically observed in the post-chemotherapy period. However, skin lesions appeared much earlier than other symptoms in the present case. A skin biopsy should always be performed in cases of cellulitis that do not respond to antimicrobial treatment to rule out any underlying causes.
